# Hyperpolarized Water for Coronary Artery Angiography and Whole-Heart Myocardial Perfusion Quantification

**DOI:** 10.3390/tomography10070084

**Published:** 2024-07-13

**Authors:** Yupeng Zhao, Mathilde Hauge Lerche, Magnus Karlsson, Rie Beck Olin, Esben Søvsø Szocska Hansen, Malene Aastrup, Mohsen Redda, Christoffer Laustsen, Lars G. Hanson, Jan Henrik Ardenkjær-Larsen

**Affiliations:** 1 Department of Health Technology, Technical University of Denmark, DK-2800 Kgs. Lyngby, Denmark; yupzh@dtu.dk (Y.Z.); mhauler@dtu.dk (M.H.L.); mkarls@dtu.dk (M.K.); rbeha@dtu.dk (R.B.O.); lghan@dtu.dk (L.G.H.); 2 MR Research Centre, Aarhus University, DK-8200 Aarhus, Denmark; esben@clin.au.dk (E.S.S.H.); malene.aastrup@clin.au.dk (M.A.); mohsen@clin.au.dk (M.R.); cl@clin.au.dk (C.L.); 3 Danish Research Centre for Magnetic Resonance, Centre for Functional and Diagnostic Imaging and Research, Copenhagen University Hospital Hvidovre, DK-2650 Hvidovre, Denmark

**Keywords:** hyperpolarized MRI, coronary artery angiography, perfusion, myocardium

## Abstract

*Purpose:* Water freely diffuses across cell membranes, making it suitable for measuring absolute tissue perfusion. In this study, we introduce an imaging method for conducting coronary artery angiography and quantifying myocardial perfusion across the entire heart using hyperpolarized water. *Methods:* ^1^H was hyperpolarized using dissolution dynamic nuclear polarization (dDNP) with UV-generated radicals. Submillimeter resolution coronary artery images were acquired as 2D projections using a spoiled GRE (SPGRE) sequence gated on diastole. Dynamic perfusion images were obtained with a multi-slice SPGRE with diastole gating, covering the entire heart. Perfusion values were analyzed through histograms, and the most frequent estimated perfusion value (the mode of the distribution), was compared with the average values for ^15^O water PET from the literature. *Results:* A liquid state polarization of 10% at the time of the injection and a 30 s *T*_1_ in D_2_O TRIS buffer were measured. Both coronary artery and dynamic perfusion images exhibited good quality. The main and small coronary artery branches were well resolved. The most frequent estimated perfusion value is around 0.6 mL/g/min, which is lower than the average values obtained from the literature for ^15^O-water PET (around 1.1 and 1.5 mL/g/min). *Conclusions:* The study successfully demonstrated the feasibility of achieving high-resolution, motion-free coronary artery angiography and 3D whole-heart quantitative myocardial perfusion using hyperpolarized water.

## 1. Introduction

Coronary artery disease (CAD) is the most common heart disease worldwide according to the World Health Organization [1]. The condition is a multifactorial phenomenon and occurs due to an inadequate blood supply to the myocardium. It is mostly caused by the formation of plaques in the lumen of the coronary arteries, which results in narrowing the blockage of these vessels, resulting in compromised and reduced blood flow or even total occlusion. Angiography can help diagnose these conditions in the macrovessels, while perfusion imaging helps diagnose reduced or blocked microvascular circulation.

Coronary computed tomography angiography (CCTA), cardiac MRI, and SPECT/PET are common noninvasive modalities for CAD diagnosis [2]. However, CCTA uses an iodinated contrast medium, and SPECT/PET uses a radioactive probe as a contrast agent, increasing radiation risk [3,4]. In contrast, MRI is a non-ionizing, mostly non-invasive technique providing coronary artery anatomy (coronary MRA [5]) and tissue perfusion information (DCE-MRI [6]). However, the image quality and resolution are limited by low SNR. DCE-MRI uses gadolinium(Gd)-based contrast agents. Some studies have shown a potential risk of gadolinium retention in the body and environmental damage caused by using gadolinium-based contrast agents [7,8].

Hyperpolarized ^13^C-labelled compounds have been employed in coronary MRA and myocardial perfusion studies to overcome the SNR limitations associated with conventional MRA and perfusion techniques [9,10,11,12,13]. Protons, compared to ^13^C, have a four times higher gyromagnetic ratio. When utilizing the same imaging gradients, proton imaging can achieve a resolution four times higher than ^13^C imaging. Compared to ^13^C, a much higher ^1^H concentration in solution can be achieved, resulting in higher SNR. Additionally, standard scanner hardware, coils, and pulse sequences can be employed for proton imaging, while special coils and sequences need to be developed for ^13^C imaging. Unlike Gd-based contrast agents, which have a large molecular size, water benefits from its small molecular size and neutral electrical charge, enabling it to freely traverse cell membranes and diffuse into tissues. Therefore, it allows for absolute perfusion quantification. For example, ^15^O water is used in PET as the gold standard for absolute perfusion quantification [14]. Considering these advantageous properties, hyperpolarized water emerges as a potential contrast agent for coronary angiography and myocardial perfusion with minimal safety concerns. Studies have demonstrated the hyperpolarization of water in a large dose using dissolution dynamic nuclear polarization (dDNP) [15,16]. High proton liquid-state polarization and long *T*_1_ relaxation times were achieved using the dDNP method with UV-generated radicals [15]. Additionally, studies also demonstrated the feasibility of performing in vivo imaging using hyperpolarized water in rat and pig [17,18,19]. In a previous coronary MRA study utilizing hyperpolarized water [18], a 5% proton liquid state polarization was achieved using dDNP with the TEMPO radical. Dynamic images of coronary arteries were acquired with a 1.3 mm in-plane resolution, without cardiac gating, resulting in image blurring due to cardiac motion.

Building on the result from the previous study, this study has three primary objectives. Firstly, we aim to polarize protons by employing dDNP with UV-generated radicals, seeking to attain a higher liquid state polarization and a longer *T*_1_ relaxation time compared to using the conventional TEMPO radical. Secondly, our goal is to further optimize the coronary MRA technique to reduce image motion artifacts and improve image resolution. Lastly, we aim to demonstrate the feasibility of 3D whole-heart quantitative myocardial perfusion using hyperpolarized water. Both coronary MRA and myocardial perfusion techniques are evaluated in a pig model.

## 2. Methods

### 2.1. Sample Preparation

A mixture of glycerol and water was used to obtain a glass-forming solution suitable for hyperpolarization using the dDNP method. Pyruvic acid (natural abundance ^13^C) was employed as a radical precursor. Sample preparation for the water hyperpolarization experiments generally followed a procedure described elsewhere [15] and briefly summarized here: Pyruvic acid (1.25 mL, 1.58 g) was mixed with a 1:1 (*v*:*v*) glycerol/water mixture (5 mL, 5.65 g). Drops (10 μL) from this mixture were pipetted into liquid nitrogen in a large dewar bowl forming solid, transparent beads. Sixty of these beads were then transferred to an EPR liquid nitrogen coldfinger placed in a custom-made holder, after which they were irradiated with UV light (365 nm, Hamamatsu LC-L5G) for (2 × 500 s) to induce the formation of the pyruvic acid radical [20]. After the irradiation was completed, the sample was investigated with EPR, showing a concentration of pyruvic acid radical of approximately 30 mmol/L. The beads were then transferred to pre-cooled cryotubes (Nunc, 2 mL) and stored in liquid nitrogen until usage. All chemicals used in the study were purchased from Sigma-Aldrich (Søborg, Denmark) and the purities of the chemicals are as follows: D_2_O (99.9% D), pyruvic acid (98%), glycerol (99.5%), Trizma^®^ base (99.8%).

### 2.2. Hyperpolarization

For each experiment, 60 beads (600 μL sample) were used. The beads were transferred from the storage cryotube to a pre-cooled 2 mL sample vial and fitted to the SpinAligner polarizer fluid path (Polarize ApS, Copenhagen, Denmark). Insertion into the polarizer followed a fast loading procedure to prevent the loss of the radical due to heating of the sample. After insertion, the sample was hyperpolarized by irradiating with microwaves at 188 GHz. The time constant for the hyperpolarization buildup was approximately 25 min. Samples were dissolved after approximately 1 h of polarization buildup.

### 2.3. Dissolution of Hyperpolarized Sample

After completing the hyperpolarization buildup (approx. 1 h), the sample was dissolved in 7.5 mL of a deoxygenated (argon bubbling) and tonicity-matched TRIS buffer, Trizma^®^ base in D_2_O solution. The water proton concentration in the dissolved sample was approximately 5 M. After dissolution, the sample was aspirated into a syringe and immediately transported to the MR-scanner room for injection. A small fraction of the sample (550 μL) was set aside and used for measuring the polarization and *T*_1_ of the water protons.

### 2.4. Animal Protocol

One healthy 40 kg female Danish domestic pig participated in the study. The animal was treated according to the Danish regulation of animal experiments (J.nr.: 2014-15-2934-01013). It was pre-sedated, and anesthesia was maintained by continuous intravenous infusion of Propofol. The animal was intubated and mechanically ventilated. A 6F catheter was placed inside the left coronary artery circumflex via X-ray guidance. The catheter placement was confirmed in the MR image after moving the pig from the operation room to the scanner bed.

### 2.5. Imaging Protocol

All imaging experiments were performed on a 3T scanner (Discovery MR750, GE Healthcare, Chicago, IL, USA).

- **^1^H Multi-phase Anatomical Images**: Acquired in a short axis view using a multi-slice 2D cine FIESTA (steady-state free precession type sequence) with breath hold. The sequence parameters are as follows: FOV = 400 × 400 mm^2^, resolution = 0.78 × 0.78 mm^2^, TE/TR = 1.5/3.4 ms, slice thickness = 10 mm, flip angle = 55°, cardiac phases = 30.
- **Hyperpolarized ^1^H Multi-frame Coronary Artery Images**: Acquired as a projection in the long axis plane of the heart with cardiac gating in diastole using a 2D gradient echo sequence with the following parameters: flip angle = 2°, TE = 2.2 ms, TR = 4.4 ms, slice thickness = 150 mm, FOV = 120 × 108 mm^2^, in-plane resolution = 0.6 × 0.6 mm^2^, acceleration = 2, heart rate = 51 bpm, frame rate = 1 frame per heartbeat.
- **Hyperpolarized ^1^H Myocardial Perfusion Images**: Acquired to cover the whole heart in the short axis using a multi-slice 2D gradient echo sequence with the following parameters: flip angle = 2°, TE = 1.4 ms, TR = 2.7 ms, slice thickness = 8 mm, number of slices = 10, FOV = 120 × 108 mm^2^, in-plane resolution = 1.5 × 1.5 mm^2^, acceleration = 2, heart rate = 59 bpm, frame rate = 1 frame per heartbeat.

Two injections of hyperpolarized water were administered in the study. One for coronary MRA and another for myocardial perfusion imaging.

### 2.6. Perfusion Quantification

Due to the short *T*_1_ relaxation time constant of hyperpolarized water in myocardial tissue (approximately 1 s at 3T [21]), the tissue signal is significantly influenced by *T*_1_ relaxation. To mitigate the impact of *T*_1_ relaxation on the estimated perfusion, a method described in [22] using only the signal upslope for quantifying perfusion, is adapted. To compensate for *T*_1_ relaxation, both the tissue and arterial signals are multiplied by an exponential function increasing with the tissue $T_{1}$ relaxation time constant, before using the upslope method as described in [22]. The mathematical justification is provided below. In addition to $T_{1}$ relaxation, the RF pulses also contribute to the decay of longitudinal magnetization. However, due to the limited number of small flip angle excitations (2 degrees) applied in each slice per frame, this effect of RF pulsing is insignificant and safely ignored in the following. However, it can easily be incorporated via the well-known concept of an effective $T_{1}$ modified by pulsing.

According to the one-compartment perfusion model, the tissue signal $S_{t}(t)$ can be modeled as the perfusion *F* multiplied by the convolution of the arterial input function $S_{a}\left(t\right)$ (the AIF) and the residue function $R\left(t\right)$ that describes tissue signal decay due to outflow. The tissue signal is proportional to the tracer concentration, which is here magnetization density. For hyperpolarized substances, *T*_1_ relaxation contributes to the signal decay in addition to outflow. Therefore, we incorporate the corresponding tissue signal decay, described by the factor $e^{-\frac{t}{T_{1t}}}$, into the one-compartment perfusion model:

$$S_{t}\left(t\right)=F\int_{0}^{t}S_{a}\left(\tau \right)\;R\left(t-\tau \right)\;e^{-\frac{t-\tau }{T_{1t}}}\;d\tau \;$$

Differences in *T*_2_ relaxation can also be included in the formulation, but were ignored here since signal decay is insignificant for the short echo times applied. This formula can be rewritten as:

$$S_{t}\left(t\right)\;e^{\frac{t}{T_{1t}}}=F\int_{0}^{t}S_{a}\left(\tau \right){\;e}^{\frac{\tau }{T_{1t}}}\;R\left(t-\tau \right)\;d\tau \;$$

This shows that the effect of relaxation can be incorporated by multiplying both the measured signal curves by a common exponential function, increasing with the tissue $T_{1}$ relaxation time constant. For the test pig, this is expected to be similar to the *T*_1_ of normal myocardial tissue, i.e., around 1 s at 3T [21] since the hyperpolarized water diffuses into normal tissue water. However, $T_{1t}$ may become longer due to the injected D_2_O. In addition to choosing $T_{1t}=1$ s for the data processing, we evaluated a range of values from 1 to 6 s to determine the sensitivity to this parameter choice.

By superimposing anatomical and perfusion-weighted images, the arterial region was found in the top slice and masked through signal thresholding. The AIF was calculated as the mean signal within this identified arterial region.

## 3. Results

### 3.1. Hyperpolarization

With a measured *T*_1_ of 30 ± 1 s at 1.4T and 28 °C, the estimated polarization of the sample at the time of injection (23 ± 2 s transfer time) was approximately 10 ± 2%. The injection volume is 7 mL, and the proton concentration was 5 M.

### 3.2. Coronary MRA

**Figure 1:**
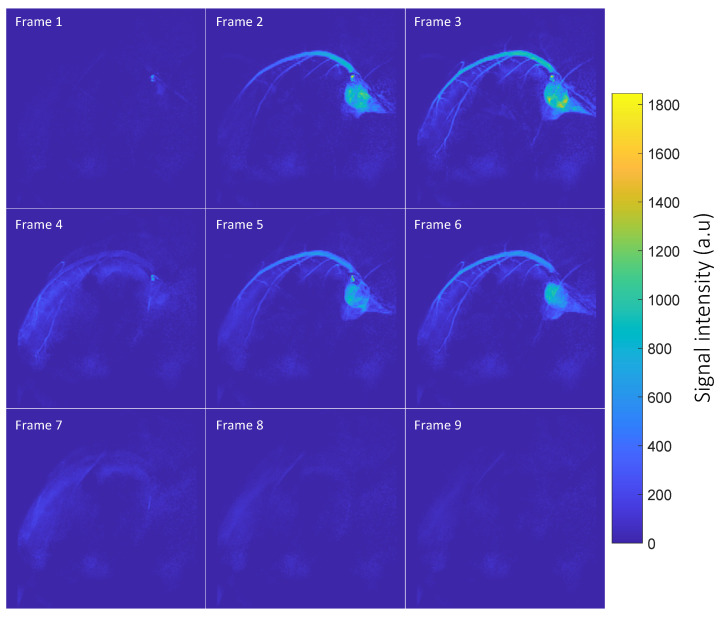
Time-resolved coronary MRA. The figure depicts the arrival of two contrast agent boluses. Frames 1–3 capture the initial arrival of the first bolus in the main artery branch, spreading to the smaller branches. Frames 4–6 show the arrival of the second bolus. Notably, both the main and small left coronary circumflex arteries are well resolved in the images.

**Figure 2:**
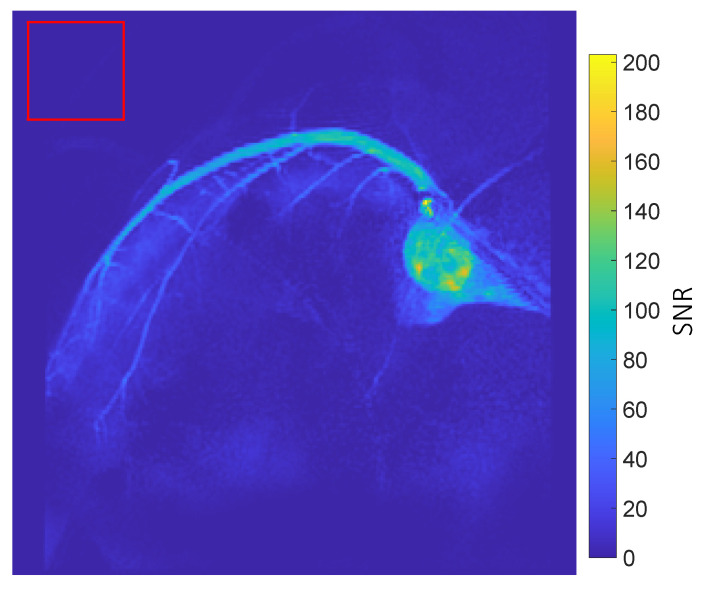
SNR map of coronary artery images: The SNR is calculated as the ratio between the signal and the standard deviation (SD) of noise. In each voxel, the signal is determined as the maximum signal intensity among all sampled time steps. The SD of noise is measured in the background (red square delineation), where no hyperpolarized signal is present. The map reveals that the coronary arteries in the image exhibit a high SNR (around 120). Additionally, the small branches at the end of the arteries also display a moderate SNR, approximately 60.

Figure 1 displays dynamic coronary MRA images, with the thermal signal removed by subtracting images acquired before injection from those acquired after injection. The figure shows the left coronary artery circumflex. Two boluses can be seen in the images. The first bolus arrives at frames 1–3. The second bolus arrives at frames 4–6 and is caused by flushing the remaining hyperpolarized water inside the injection tube using saline. Figure 2 displays the SNR map of coronary artery images. In general, the coronary arteries exhibit high SNR. The map illustrates that the main branch of the coronary arteries has a high SNR, approximately 120, while the smaller vessels at the end of the main coronary artery branch show a moderate SNR, around 60.

### 3.3. Myocardial Perfusion Quantification

**Figure 3:**
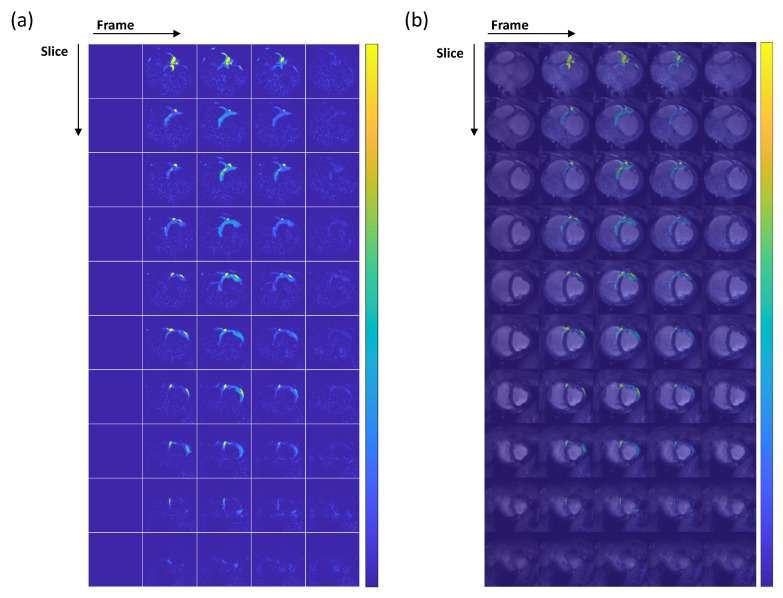
(**a**) Dynamic perfusion-weighted images: The contrast agent arrival starts at frame 2. Notably, frame 2 reveals a high-amplitude signal in the main artery (slice 1), and the contrast agent perfuses the heart tissues in the subsequent frames. However, due to the short *T*_1_ relaxation time of hyperpolarized water in blood, the increased signal is visible only over four frames. (**b**) Overlay of perfusion and anatomical images. The myocardial region perfused by the hyperpolarized water can be seen in the figure. The colorbar in both subfigures (**a**,**b**) covers the signal range.

Figure 3 depicts myocardial perfusion images where, similar to coronary MRA, the thermal signal has been removed. These images cover the short axis of the heart from base to apex. Due to the placement of the catheter in the left coronary artery circumflex, only a portion of the myocardium is perfused with hyperpolarized water, supplied by the left coronary artery circumflex. The perfused segment of the myocardium is visible in Figure 3a. Figure 3b illustrates the overlay of perfusion images on thermal proton anatomical images. In this figure, the region of the myocardium supplied by the left coronary artery circumflex can be identified as the area with increased signal in the overlaid anatomical images. The signal initially appears in the main arteries and then reflects perfusion into the myocardial tissue (the signal shows up in the myocardium starting from frame 2). After injection, the *T*_1_ relaxation time of hyperpolarized water gradually decreases from 30 s in TRIS buffer to 1.6 s in normal blood at 3T [23], resulting in signals that last only over four frames.

**Figure 4:**
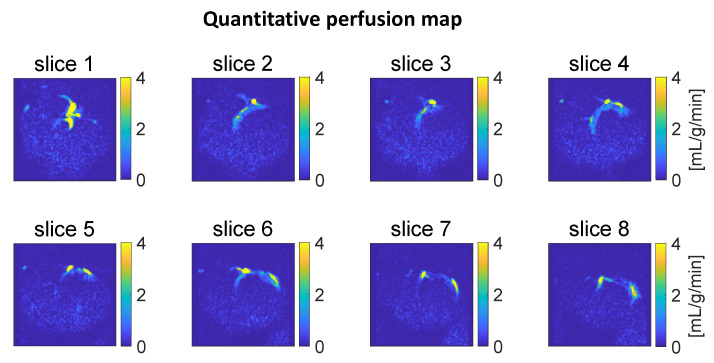
Quantitative perfusion maps obtained from the upslopes of the signal time courses. Slices 1–8 depict the heart from base to apex. The yellow areas indicate artifactual high perfusion values in the coronary artery.

**Figure 5:**
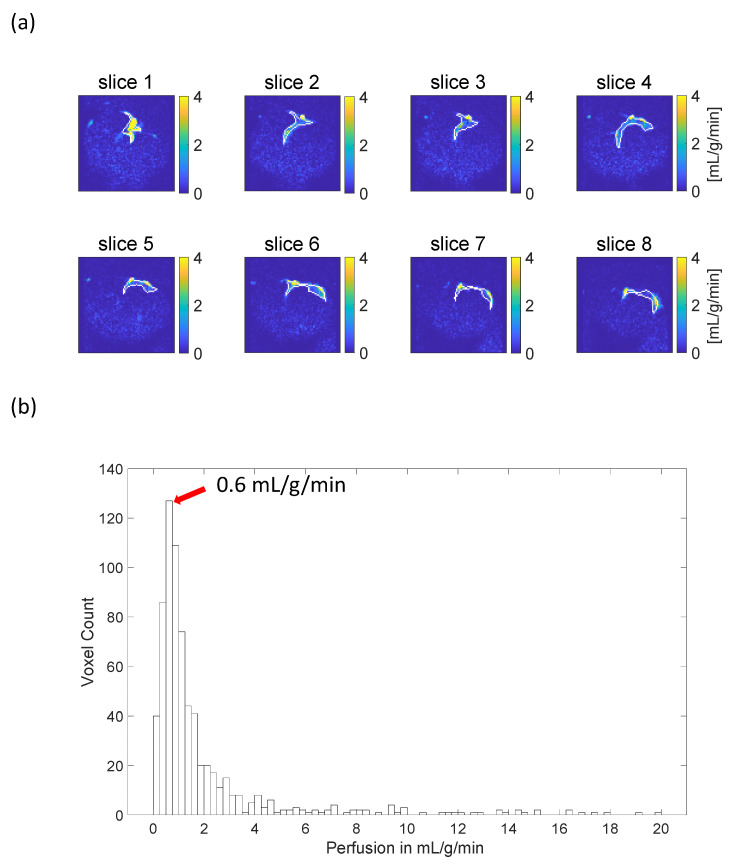
Histogram of estimated perfusion values within a region exhibiting high signal intensity. The region was delineated manually based on dynamic perfusion-weighted images to encompass both arterial and tissue regions. In (**a**), quantitative perfusion maps are shown, and the area outlined by the white line represents the manually drawn signal region. In (**b**), the perfusion values within this region are analyzed using a histogram. The most frequent estimated perfusion value is around 0.6 mL/g/min, which is lower than literature values [24,25] (around 1.1 and 1.5 mL/g/min). Much higher estimated values appear due to arterial signal contributions (yellow regions in Figure 3).

Figure 4 shows the quantitative perfusion map, and Figure 5 illustrates the histogram of estimated perfusion values within the region of increased signal. The results are calculated with the $T_{1t}^{\;}$ being set to the normal tissue $T_{1}^{\;}$ relaxation time (1 s at 3T [21]). The signal region is manually delineated based on the superimposed anatomical and perfusion-weighted images. It is indicated by the white line shown in Figure 5a. The mode of the distribution is around 0.6 mL/g/min, which is lower than two literature average values estimated using PET (^15^O-water), 1.1 and 1.5 mL/g/min [24,25].

In addition to choosing $T_{1t}=1$ s for the perfusion quantification as argued earlier, a range of $T_{1t}$ values from 1 to 6 s were used in a repeated analysis to evaluate the sensitivity to this parameter choice. The analysis showed that the perfusion estimates depend on the assumed $T_{1t}$ value. The most frequent estimated perfusion value increases from 0.6 mL/g/min to 1.1 mL/g/min, with the $T_{1t}$ value increasing from 1 to 3 s. Further increasing the $T_{1t}$ value does not significantly change the perfusion estimates.

## 4. Discussion

This study builds on a previous study [18] and introduces several notable improvements. Firstly, the image resolution was doubled from 1.3 mm to 0.6 mm in two dimensions, and the fine vessels at the end of the artery branches were clearly resolved. Secondly, the method introduced a solution to mitigate cardiac motion artifacts by employing cardiac gating. Thirdly, UV-generated radicals proved effective in achieving higher liquid-state polarization levels compared to TEMPO radicals. Significantly, this study is the first to demonstrate the feasibility of a 3D whole-heart quantitative myocardial perfusion estimation using hyperpolarized water, with the most frequent estimated perfusion value (the mode of the distribution) somewhat lower than the PET (^15^O water) literature average values [24,25]. The difference between the mode of the distribution and the average values from the literature can have several causes. Firstly, the two values have different meaning, making direct comparison suboptimal. The large, analyzed region, which includes both tissue and arterial sections, skews the average value due to the high estimated perfusion in the arterial region, rendering it unrepresentative of the average tissue perfusion. This makes the chosen approach necessary. Secondly, the literature value represents the average of the entire myocardium, whereas the mode of the distribution in this study is derived from a segment of the myocardium. This difference in analyzed regions can result in bias. Thirdly, the tissue $T_{1}$ relaxation time constant in this study was set to the human myocardium $T_{1}$. The actual tissue $T_{1}$ for the particular pig may differ from the set value, introducing additional bias. Similarly, the perfusion of the particular pig may actually differ from the literature sample averages. Lastly, the injected sample contains relatively high concentrations of glycerol and pyruvate, which potentially have physiological effects on tissue perfusion.

A related point to consider is the contribution of the hyperpolarized signal from different chemicals. The protons from water, glycerol, and pyruvate all contribute to the hyperpolarized signal. The proton concentration ratio in the sample is approximately 70% from water, 26% from glycerol, and 4% from pyruvate. The $T_{1}$ relaxation times of glycerol and pyruvate are expected to be similar to those of water because both glycerol and pyruvate are small mobile molecules, as are choline, creatine, and N-acetylaspartate, which are known to have proton $T_{1}$ values similar to those of water in vivo (a few seconds, at most). Therefore, the signal is expected to be dominated by hyperpolarized water.

The current approach for coronary MRA is limited to 2D projection imaging, whereas state-of-the-art techniques utilize a 3D imaging approach with an isotropic millimeter resolution. It is worth noting that 3D coronary MRA can be implemented using a cardiac-gated 3D multi-frame sequence. Secondly, the short *T*_1_ relaxation time necessitates direct contrast agent delivery to the heart tissue, restricting its broader clinical application. Thirdly, the current catheterization approach involves placing the catheter inside the left coronary artery, making the measurement of perfusion in the entire myocardium impossible. To measure perfusion values in the entire myocardium, the catheter must be positioned either in the left ventricle or in both the left and right coronary arteries and multiple injections would have to be made. Future improvements, such as enhancing polarization levels and increasing injection volumes, may pave the way for intravenous injection, potentially making the method applicable in clinical settings.

## 5. Conclusions

The study successfully demonstrated the feasibility of achieving high-resolution, motion-free coronary artery angiography and 3D whole-heart quantitative myocardial perfusion using hyperpolarized water. The results indicated that hyperpolarized water serves as a suitable contrast agent for both coronary MRA and myocardial perfusion. However, further studies are necessary to assess the reproducibility of the methods and to compare their performance with state-of-the-art techniques.

## Data Availability

Data are available upon request. Please contact the corresponding author, jhar@dtu.dk.
